# The role of peripheral nerve surgery in a tissue reinnervation

**DOI:** 10.1186/s41016-019-0151-1

**Published:** 2019-02-18

**Authors:** Alexander O. Tuturov

**Affiliations:** Department of Physiology, Samara State Medical University, Samara, Russian Federation

**Keywords:** Neurotization, Nerves, Conduit, Reinnervation, Neurorrhaphy

## Abstract

In modern neuroscience, the most relevant is the study of the problem of reinnervation of tissues after severe injuries. Complete restoration of lost physiological functions is still impossible with lesions of peripheral nerves with the formation of extensive diastasis between their proximal and distal sites. In this case, the standard neurorrhaphy cannot be carried out because of the eruption of the filaments during tension and convergence of the ends. To solve this problem, a technique was developed for autotransplantation of the nerve sections, which is still *the gold standard* for the reconstruction of extensive nerve defects. However, the presence of significant shortcomings led to the development of the doctrine of the direction of regeneration with the help of conduits. Currently, the use of nerve channels is the most promising technology for peripheral nerve repair after trauma.

The most actively developing now is the direction of reinnervation, such as neurotization. Neurotization, in some way, combined all the methods of restoring nerves.

The overall goal of all these methods—the restoration of extensive nerve defects—allows them to be combined into a new industry: reinnervating neurosurgery.

## Background

The loss of afferent and efferent innervation of tissues due to mechanical injuries, oncologies, and other damaging factors is a serious medical problem. There are two etiological variants of nervous system damage: central and peripheral. These different pathological mechanisms have a common goal of treatment: complete or partial reinnervation.

**Fig. 1:**
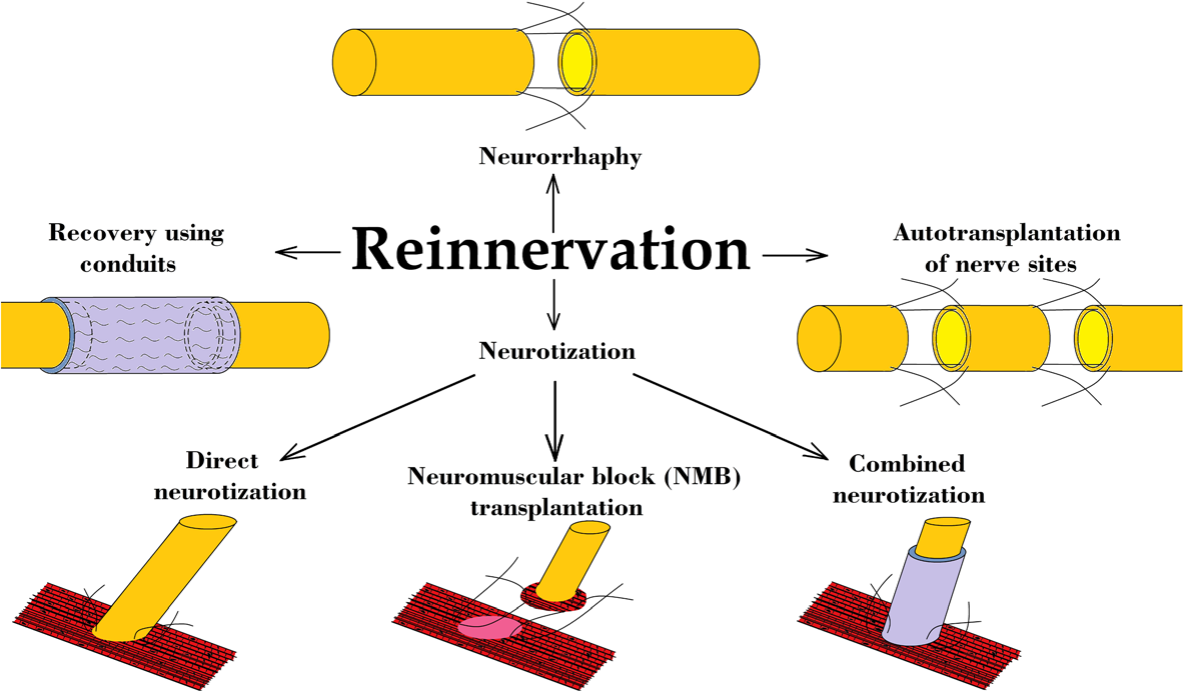
Schematic representation of the methods of reinnervation of tissues in neurosurgery

Reinnervation of tissues includes several techniques (Fig. 1):

- Neurorrhaphy
- Autotransplantation of nerve sites
- Recovery using conduits
- Neurotization

Each of the methods has its own indications and contraindications. It is most interesting to consider these surgical techniques in case of damage to the central nervous system, for example, the spinal cord. Every year in the world from 8 to 250 cases per million inhabitants account for spinal cord injuries, with more frequent harms to its cervical spine (about 50%) [1]. Damage to this area of the central nervous system leads to severe impairment of motor functions, such as di- or tetraplegia, suppression of diaphragm function, and other dysfunctions. Damage to segments of the thoracic and lumbar spinal cord can lead to persistent dysfunction of the pelvic organs and lower extremities, which are also difficult to treat and rehabilitate [2]. In these cases, it is necessary to use variants of reinnervation to bypass the damaged segment. That is the redirection of the nerve impulse from the working zone of the spinal cord to the denervated nerve trunks.

Lesions of the peripheral nervous system are most often associated with motor dysfunction. The result is a permanent loss of motions with the development of paralysis and muscle atrophy accompanied by the formation of contractures [3]. In these cases, any variants of reinnervation can be applied. However, there is a dependence by the clinical situation and the possibilities of surgical technique.

## Neurorrhaphy

Neurorrhaphy or nerve suture is the main method of recovery. Probably, the first time it was stated by Claudius Galen. He considered surgical restoration of tendons in his work *Ars parva*. However, this is conditional, because at that time Hippocrates, Galen, and Avicenna did not notice a significant difference between tendons and nerves. So, they determined a common tactic of these surgeries [4]. After several centuries, the tendons were differentiated from nerves by Albert van Heller. Thus, he approved the tenorrhaphy, but he distanced the doctors from the nerve suture. Because of this, there was a fear of causing causalgia at the slightest touch to the *bare* nerve tissue. Until now, there is no indication of a particular researcher, who discovered the era of peripheral nervous system surgery [5].

Now, there are several types of nerve seams, differentiated by the suture option: epineural and perineural (fascicular). It is most interesting to consider variants of anastomosing because at the present time, there is one significant difference between the technique of nerve microsurgery and the historically accepted concept: the variability of the regeneration vector. The different zones of nerve anastomosis denote three types of nerve sutures: end-to-end (ETE), end-to-side (ETS), and side-to-side (STS). The first two methods are most studied and are more often used in neurosurgery of the peripheral nervous system, than the third one [6].

At the same time, ETS anastomosis is still being criticized, but less than STS [7]. It is believed that the ETS has one key advantage: the possibility of using the proximal functioning end of the nerve in the inaccessibility or complete dysfunction of the distal [8]. This makes the technique more accessible for reinnervation of denervated tissues. In addition, Lundborg in his works not once pointed out that, perhaps, an ETS nerve seam is a more universal and promising method of neurorrhaphy [9]. Other researchers also came to understand this concept after the development of the theory of termino-lateral regeneration of the nerve and the possibilities of its collateral sprouting. This served to form the idea that the suture STS has more opportunities than the ETS. Due to the fact that with this technique, a collateral sprouting of the nerve is better provided [10].

Because of the contradiction of opinions, it is important to conduct an analysis to determine the effectiveness of each method. Ronkko’s research is devoted to comparing the effectiveness of these three operational techniques.

The experiments were performed in rats. Test groups included:

I. The intersection of the common peroneal nerve with subsequent anastomosing of the proximal and distal ends (ETE);
II. The formation of an epineural window in the tibial nerve and anastomosing the common peroneal nerve (ETS) to the epineural window;
III. The formation of epineural windows in the common peroneal nerve and tibial nerve 2 mm in diameter and anastomosing between them (STS).

Suture material 10/0 Nylon and four epineural seams were used. The control group is the fourth series, in which the nerve suture was not performed.

Experimental groups showed signs of restoration of neuroconductivity. However, groups II and III did not differ significantly in the course of functional, morphometric, and histological studies. The most preferred technique was in group I (ETE), which was better in all indicators for 6 and 26 weeks. The results at 26 weeks show a difference in the area of the nerve fiber—36,779 n/mm^2^ (group I), 19,554 n/mm^2^ (group II), and 16,653 n/mm^2^ (group III) [11].

The high efficiency of neurorrhaphy in ETE and ETS types was experimentally confirmed. Probably, correction of suture technique can improve these indicators.

## Autotransplantation of nerve sites

One of the most difficult problems encountered on the path to successful nerve regeneration is the posttraumatic defect of the nerve trunk. Diastasis after lesions prevents comparison of nerve sites, promotes neuroma development, and also depresses the nutrition of distal end tissues [12].

Researchers developed a technique for autotransplantation of sections from a nerve donor to a nerve recipient. This ensured the replacement of diastasis. Next, an ETE seam was performed, which restored integrity after extended defects. The authors of this technique are also several researchers: J.M. Philipeaux, A. Vulpian, Albert, H. Millesi, and Seddon [5]. Like the classical neurorrhaphy, the seam of the autograft has not yet undergone any significant changes. At the same time, it is now considered to be the *gold standard* for the recovery of nerves with a post-traumatic extended defect. This technique is successfully used to reinnervate the diaphragm, the biceps brachii, and other muscles [13].

On the other hand, the present method has limited application. Currently, this technique has been criticized because it had negative long-term results. Disadvantages are the formation of neuroma in the places of the graft suture, partial or complete loss of sensitivity, and the probability of rotation of the bundles of nerve fibers in different planes. At the same time, a significant disadvantage of autotransplantation is the need for additional surgery and limited choice for tissue collection. Often, the diameters of the donor nerve and the recipient’s nerve are different due to the anatomical features of the location [14].

Because of this, the methodology did not become final. Further development contributed to the creation of new technology for the restoration of extended nerve defects, which involves the use of conduits instead of autografts.

## Recovery using conduits

The idea of creating *a bridge* between the intersected parts of the nerve appeared in the XIX century when T. Gluck applied decalcified bone in 1880 as a hollow conductor for regeneration [6]. Since then, a new era of stimulation of neural regeneration has begun. At the same time, the notion of a conduit was established. This is a conductor of cylindrical shape, which is used to repair posttraumatic extensive nerve defects. He directs the regeneration of nerve fibers in his cavity from the proximal end of the nerve trunk to the distal one.

Later, the area of the brachial artery began to be used as conduits, then the autovein [12]. However, they were also accompanied by a number of shortcomings: a restriction in the selection of the required diameter, the absence of suitable vascular donors, the decline of the venous wall during regeneration, and others [15]. To eliminate these problems, the search direction for the necessary conduit was changed, which led to the creation of artificial conductors.

Now, there are reports of the use of various materials and substances that are capable of directing nerve fibers [16]. An essential advantage of the nerve conduits of the XX and XXI centuries is the presence of an inner environment in their cavity, which has a stimulating effect on the regeneration of the axons and also prevents the formation of a neuroma [12].

Requirements were made for an ideal conduit in order to form a refined concept for the study of materials and forms [17]. Now, the most relevant of them is the ability to biocompatibility and biodegradation, as well as ease of manufacture and use. In addition, it was proved that the ideal conduit should contain microchannels for accurate matching of nerve fibers [18].

Now, the technology of restoring nerves with conduits is the most promising method than using autografts. However, conduits and their inner environments are still not ideal. No conduit technology reports a complete restoration of the integrity of the nerve, a comparison of all nerve fibers with each other and the return of all lost functions [19].

The presented methods of nerve repair after damage are not fully effective. However, a new direction of neuroregeneration is now developing—neurotization, which is the consensus of all traditional methods of peripheral nervous system surgery.

## Neurotization

Neurotization or the method of direct muscle reinnervation is the least studied, but at the same time, the most promising way to restore post-traumatic denervation. The application of the technique is justified when neurorrhaphy is not possible due to the absence or serious damage of the distal end of the nerve. In this case, autotransplantation and the use of conduits are impossible. For reinnervation in such cases, the proximal end of the nerve is directly implanted into the muscle tissue.

The first mention of successful neurotization refers to 1908 when the Hacker restored the motor function of the trapezius muscle of a 12-year-old patient. He implanted in the structure of this muscle the proximal segment of the accessory nerve along with the motor branch from the cervical plexus [20]. However, despite the success, neurotization has not been used for a long time and has found application only in experimental studies on animals.

The problem of formation of new terminal plates or neuromuscular junctions (NMJ) attracts the greatest interest in this direction of neuroregeneration. The NMJ is the zone, in which the nervous system through the peripheral nerves interacts with the fibers of skeletal muscles and forces them to contractions. Damage to this NMJ mechanism causes many genetic diseases [21].

It is necessary to form the NMJ at the morphological level to realize electrophysiologic processes. Every branch of a distal end of the nerve have the postsynaptic folds, which increase the surface area of the postsynaptic membrane. There are vesicles containing neurotransmitters, which organize the intensity of nerve transmission through ion channels to the myocytes [22].

However, NMJ is formed not only with the formation of neuromuscular contact. Agrin binds to low-density lipoprotein, which is bound to the Lrp4 receptor. Thus, a muscle-specific kinase is activated in postsynaptic muscle fibers. At the same time, the presented chain of events is relevant not only for the formation of NMJ but also for its maintenance [23].

In summary, the key task of neurotization is the formation of new NMJs or the expansion/activation of the old NMJ zone after nerve implantation.

Controversial results of many experimental works were resolved in the technology of Sobotka and Mu [24]. The authors of this study performed a dissection and coagulation of the nerve ends, innervating the left sternocleidomastoid muscle. On the opposite muscle, the distal part of the nerve trunk was resected with a site of innervated muscle tissue 6 mm × 6 mm × 3 mm in size to preserve the end plates. This neuromuscular block (NMB) was implanted by nerve suture with nylon 10/0.

Three months later, researchers stained the neurofilament with silver nitrate impregnation, and as a result, they noted that the regenerating axons from the implanted NMB sprouted into the recipient’s muscle. At the same time, the muscle mass was 87% of the control group, and the degree of functional restoration of the innervation was 66% when measuring the maximum contraction force.

Kang Sung-Bum with a group of researchers managed to restore the function of denervated muscle tissue through direct neurotization [25]. Despite the success, the authors of the paper asked the same question of the absence of an appropriate nerve site for anastomosing and implantation. Indeed, many injuries lead to serious nerve damage with the formation of extended defects. Kang et al. refused to use the autograft of the nerve, fearing the characteristic complications for them [26]. To solve the problem, the researchers applied a combined method: prolongation of the proximal nerve region with the help of a conduit for subsequent neurotization, as a material for conduit used silicone. After resection of the branches of the sciatic nerve from the innervated muscles, a conductor was placed in the resulting diastase. Its proximal end was sutured with a sciatic nerve, and the distal end was sutured with epimysium near the denervated terminal plate. The silicone conduit was filled with collagen gel. Control groups were completely denervated muscles.

In the course of the study, a stable contracture was observed in all control groups, except for *the neurotization-conduit* group. After 8 weeks in the experimental group, a weak contractile response to neurostimulation was observed, and by the end of the 20th week, the muscle response was close in strength and amplitude to normal. Histologically, in all control groups, fat infiltration and a decrease in the size of sarcomeres were observed; however, in *the neurotization-conduit* group, muscle fibers were restored, and the diameter of the axon increased. In conclusion, the authors of the study noted, that the presented combined technique can lead to a complete restoration of functions, but it is necessary to use a more sophisticated conduit and its inner environment [27].

## Conclusion

The development of tissue reinnervation technologies is necessary for progress in the rehabilitation of patients after severe denervation and tissue loss. The priority task is to provide the motor function of the muscles. However, not only efferent innervation should be the main one. Modern reconstructive surgery should strive to provide tissue with afferent nerve fibers to restore sensitivity [28].

At the present time, tissue engineering techniques are also being developed that allow artificially recreating remote sites of organs and tissues. In this regard, reinnervation of the muscular and skin grafts is important for the replacement of defects after extensive resection operations in oncosurgery and gross posttraumatic tissue defects [29]. At the same time, on the way to creating innervation, the problem arises the formation of NMJ. Moreover, in the case of tissue engineering, this issue is most relevant, since the end plates can in principle be absent, and it will not be about activation of the old ones or expansion of their coverage, but about the formation of NMJs in completely new localizations. In such a case, a well-developed technology for direct neurotization or NMB transplantation from donor sites is needed.

Research of recovery of peripheral nerves and replenishment of lost innervation of the tissues is an important step in the development of modern neuroscience. The common goals and ways of solving the tasks indicate the close connection of these problems and allow to form a new specialty—reinnervating neurosurgery.

## Abbreviations

ETE: End-to-end
ETS: End-to-side
NMB: Neuromuscular block
NMJ: Neuromuscular junction
STS: Side-to-side
